# Secondary use of health records for prediction, detection, and treatment planning in the clinical decision support system: a systematic review

**DOI:** 10.1186/s12911-025-03021-8

**Published:** 2025-05-16

**Authors:** Dipendra Pant, Øystein Nytrø, Bennett L. Leventhal, Carolyn Clausen, Kaban Koochakpour, Line Stien, Odd Sverre Westbye, Roman Koposov, Thomas Brox Røst, Thomas Frodl, Norbert Skokauskas

**Affiliations:** 1 Department of Computer Science, Norwegian University of Science and Technology, Trondheim, Norway; 2 The University of Chicago, Chicago, Illinois USA; 3 Regional Centre for Child and Youth Mental Health and Child Welfare (RKBU Central Norway), Department of Mental Health, Faculty of Medicine and Health Sciences, Norwegian University of Science and Technology, Trondheim, Norway; 4 Department of Child and Adolescent Psychiatry, Clinic of Mental Health Care, St. Olav University Hospital, Trondheim, Norway; 5 Regional Centre for Child and Youth Mental Health and Child Welfare (RKBU North), UiT The Arctic University of Norway, Tromsø, Norway; 6 Department of Computer Science, UiT The Arctic University of Norway, Tromsø, Norway; 7 RWTH University of Aachen, Aachen, Germany; 8 Vivit AS, Trondheim, Norway

**Keywords:** Secondary use, Electronic health record, Clinical decision support, Systematic review

## Abstract

**Background:**

This study aims to understand how secondary use of health records can be done for prediction, detection, treatment recommendations, and related tasks in clinical decision support systems.

**Methods:**

Articles mentioning the secondary use of EHRs for clinical utility, specifically in prediction, detection, treatment recommendations, and related tasks in decision support were reviewed. We extracted study details, methods, tools, technologies, utility, and performance.

**Results:**

We found that secondary uses of EHRs are primarily retrospective, mostly conducted using records from hospital EHRs, EHR data networks, and warehouses. EHRs vary in type and quality, making it critical to ensure their completeness and quality for clinical utility. Widely used methods include machine learning, statistics, simulation, and analytics. Secondary use of health records can be applied in any area of medicine. The selection of data, cohorts, tools, technology, and methods depends on the specific clinical utility.

**Conclusion:**

The process for secondary use of health records should include three key steps: 1. Validation of the quality of EHRs, 2. Use of methods, tools, and technologies with proactive training, and 3. Multidimensional assessment of the results and their usefulness.

**Trial Registration:**

: PROSPERO registration number CRD42023409582

**Supplementary Information:**

The online version contains supplementary material available at 10.1186/s12911-025-03021-8.

## Background

The availability of vast amounts of digital health data and technological advances have created a significant revolution in the healthcare industry. Electronic Health Records (EHRs) have become invaluable databases of patient data, containing a wide range of clinical information, procedures, and outcomes [1]. EHRs describe a patient’s medical history, diagnosis, treatments, outcomes, and other pertinent information, such as the patient’s family and social support. They give clinical practitioners a thorough understanding of the patient’s health history, which guides treatment decisions and promotes coordinated care [2].

In addition, health information exchanges allow information to be shared among different providers, ensuring that patients receive consistent treatment and care [3]. EHRs are comprehensive collections of individual health and medical information over time, including clinical data, treatment, procedures, and other relevant information, primarily electronic and also includes paper records [4]. Both electronic and paper records today serve as a central repository and are a more comprehensive and inclusive collection; therefore, we have used EHRs throughout this review of existing literature. The term Electronic Health Records or its abbreviation EHRs is used to refer to all forms of health data, including health records, electronic medical records, and observational data. The terms EHRs, health data, medical records, health information, and medical information have different meanings. However, they all are closely related in the context of health care [5]. All these are raw material for performing secondary use in health informatics.

According to the World Health Organization (WHO) [6], secondary use of EHRs refers to the processing of EHRs for purposes other than those for which they were originally collected and is helpful for decision making, research, and the management of health systems. Although the primary goal of EHRs is to support patient care, their secondary use for research and decision support has emerged as a viable way to improve health care quality. The use of EHRs for data-driven, computer-assisted decision making in the healthcare industry has recently received much attention [7].

The secondary use of medical records is significant for clinical decision support through prediction [8], detection, and treatment recommendation [9, 10] in healthcare. For the secondary use of these ever-growing and already stored vast amounts of EHRs tools, technology, and computational power, evidence-based practice guidelines, and domain experts are needed. Currently, there are the technology, tools, computing power, vast amount of EHRs, and evidence-based practice guidelines, so it is the right time. Predictive analytics, early symptom detection, and improved treatment techniques using the data stored in EHRs have the potential to transform healthcare [11]. Medical researchers and healthcare providers may be able to better anticipate, identify, and treat various medical conditions by utilizing the wealth of information contained in these records. The secondary use of data will help shed light on these.

Clinical Decision Support Systems (CDSSs) are the blueprint that makes this possible. As a result, the use of and interest in CDSSs are growing [12]. CDSSs are computer-based systems that use information and communication technologies to make relevant knowledge available for a patient’s health care and well-being [13]. Correspondingly, the secondary use of EHRs is a more focused approach to produce direct clinical utility [6, 14].

This study focuses on the secondary use of EHRs for prediction, diagnosis, classification, treatment recommendation, and related tasks for clinical utility. Part of secondary use may include secondary analysis, which focuses more on reanalysis of the EHRs, an intermediary that can contribute to the performance of secondary use through data preparation, processing, statistical analysis, and selection of appropriate methods [15], which this review has not touched upon. The motivation for the review was the Individualized Digital DEcision Assist System (IDDEAS) project, which focused on developing and implementing a clinical decision support system for Child and Adolescent Mental Health (CAMH) [16].

### Objective

The objective of the review is to understand how to perform secondary use of EHRs for prediction, diagnosis, classification, treatment recommendation, and related tasks for clinical utility. Hence, providing comprehensive knowledge of how secondary use of any EHRs can be performed in a data-driven and continuous computational manner for various clinical applications, including somatic and mental health. By conducting a systematic review, we hope to find pertinent papers that address the issues, goals, processes, and outcomes of implementing EHRs reuse. The study compares findings, assesses their therapeutic utility, and evaluates the impact of secondary data use across different clinical scenarios. It addresses the methodologies, technologies, and results of secondary data usage, aiming to consolidate existing knowledge and highlight areas needing further investigation. Finally, the review will help us to understand the possible ways, benefits, difficulties, and other issues related to the secondary use of EHRs in CDSSs.

## Methods

This systematic review follows the Preferred Reporting Items for Systematic Reviews and Meta-Analyses (PRISMA) [17] guidelines.

### Search strategy

Articles were identified from five databases, out of which two were life sciences and biomedical databases, the Scopus and PubMed, and three were the computer science databases, the ACM Digital Library, IEEE Xplore, and DBLP. The search focused on four dimensions: data, secondary use, utility, and providing support and decision making. Based on the four dimensions, the four different sets of keywords were:

- *1: “health data” OR “health record” OR “Electronic health record” OR “Electronic medical record” OR “Observational data” OR “EHR” OR “EMR” OR “medical record”*
- 2: “secondary use*” OR “secondary application*” OR “secondary analy*” OR “health* reuse” OR “clinical reuse” OR “secondary usage”
- *3: “diagnosis” OR “detect” OR “identify” OR “recognize” OR “treatment” OR “predict” OR “prognosis” OR “progress” OR “develop” OR “onset” OR “assessment” OR “management”*
- 4: “decision making” OR “decision-support” OR “decision support” OR “decision system” OR “computer* decision” OR “computer-aided decision” OR “computer aided decision” OR “DSS” OR “computer assisted decision making” OR “computer-assisted decision making” OR “clinical decision support*” OR “clinical-decision support*” OR “cds”.

Date, language, and publication status were not included as additional search restrictions. The first search was performed on March 19, 2023. The second search was conducted based on the team feedback on April 27, 2023, including additional keywords “observational data”, “clinical reuse” and “secondary reuse”, “develop”, “onset”, “assessment”, “management”. The first search yielded twelve out of three hundred seventy-five that met the inclusion criteria [18–29], and the second search yielded one more (13 out of 391) [30]. Inclusion criteria for study selection consisted of original research, peer-reviewed, in English, on the use of EHRs for decision support, including secondary use of EHRs or observational data, development and use of a computerized or electronic decision support system, secondary use for prediction, detection, and treatment recommendation, and related tasks. In parallel, the exclusion criteria included articles that did not address the secondary use of EHRs for decision support. Articles focusing solely on the primary use of EHRs and studies involving statistical and secondary analysis were excluded. The inclusion and exclusion criteria and other preliminary information were registered in PROSPERO.

### Study selection

Study selection was done using the tool *rayyan.ai* [31]. Three hundred and ninety-one records were identified from the database search. During the search, one author independently performed the search and records identification, which other authors verified. Inclusion of studies was performed by the first author with verification by the second and other authors. All authors were involved from start to end, including finding the appropriate keywords, setting objectives and criteria, setting questions for the study, and also for verification and selection. Duplicates were removed using *rayyan.ai* duplicate detection.

The keyword-based filtering was done through the union of keywords; in the record screening phase, records were excluded if the title or abstract did not contain any of the keywords. This was followed by layering-based intersection consisting of the intersection of keywords in three layers; in the reports sought for the retrieval phase, records were excluded if the title or abstract did not contain at least one keyword in all three categories/layers. Categories/layers were (1) data or record or information, (2) secondary use/usage or observational data (3) predict or detect or treatment or management or identify or develop or assessment or prediction or detection or progress or prognosis or onset or diagnose or recognize.

**Fig. 1:**
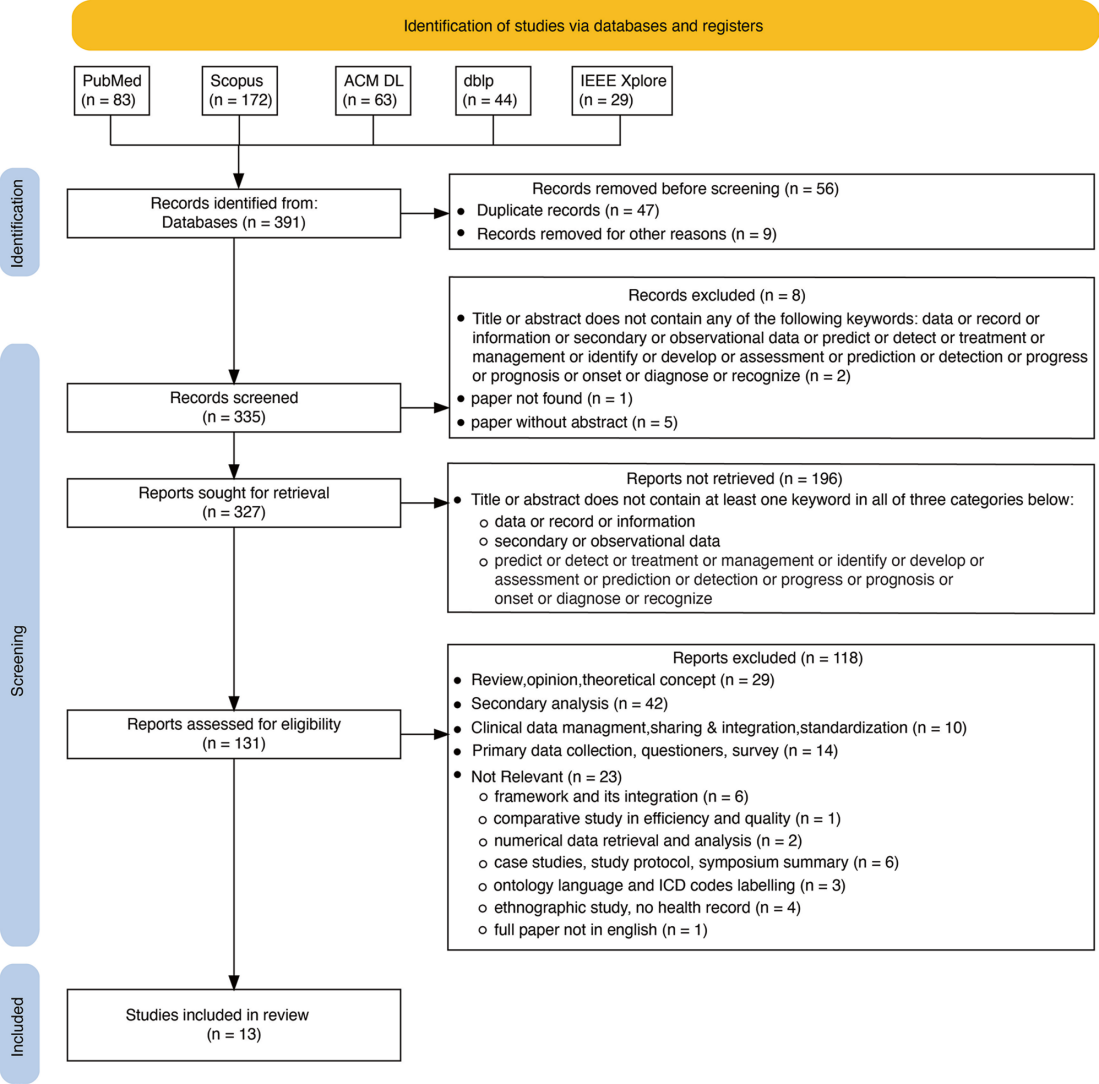
PRISMA flow diagram for the study selection

A full text study of reports assessed for eligibility was done to ensure the presence of actual secondary use implementation on EHRs for clinical prediction detection or treatment and related tasks. Articles solely focused on clinical data quality, management, system integration, labeling, and numerical analysis were excluded. The PRISMA flow diagram (Fig. 1) below describes the study selection and rationale.

### Information retrieval strategy

**Fig. 2:**
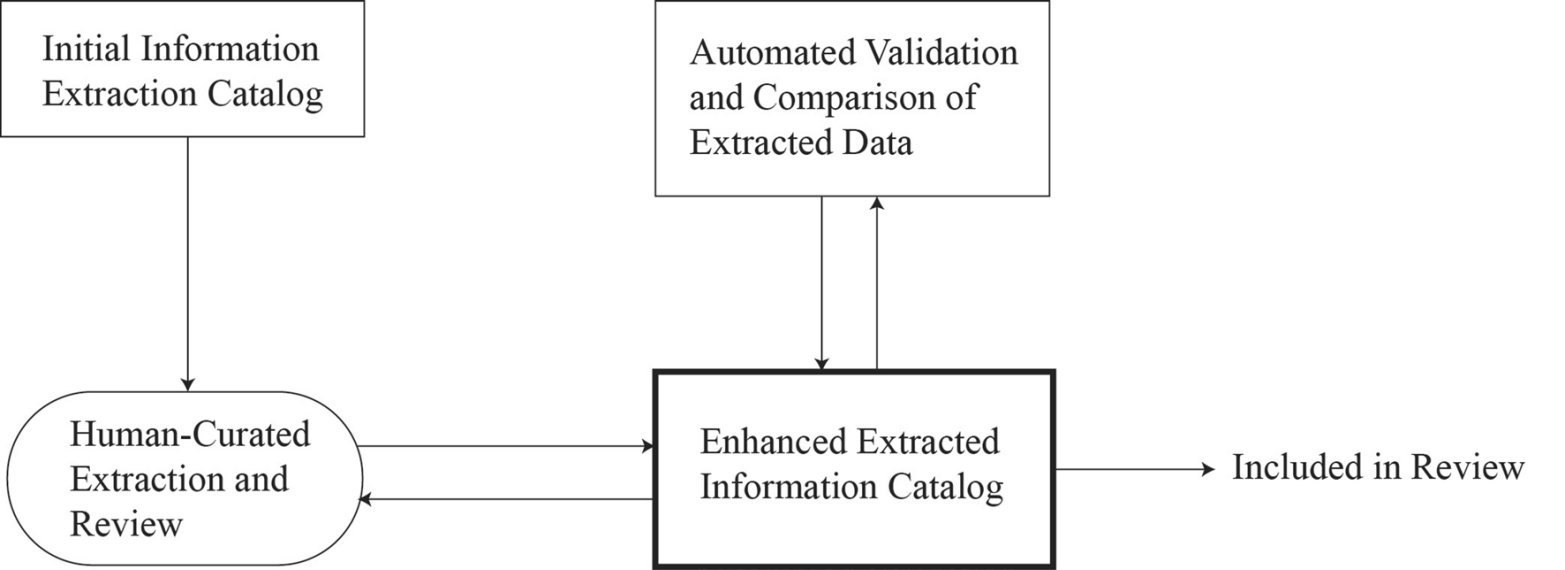
Illustration of information retrieval strategy from included studies

We followed the steps below for the information retrieval (Fig. 2). The first task was to determine what information to extract from all the selected papers, which we already had in the form of a catalogue after completing the selection of studies with PRISMA guidelines (Fig. 1) above. Given the initial catalogue of information to be extracted, the papers were reviewed by the first author, and in case of doubt, the second author and, if necessary, the team of authors were consulted. Through human-curated extraction and an in-depth review of the papers, more information was extracted, and the initial catalogue was expanded. A comparison and validation were then performed using the automated tool Elicit [32]. We found Elicit to be efficient and accurate to a certain extent for information retrieval. Thus, the retrieved information was included in the review; the summarized information is presented in the results section below.

## Results

Table 1 below summarizes focus areas, data, tools, techniques, technologies, and utilities. All these EHRs were not necessarily stored and collected with research purposes in mind; rather, their secondary use was research purposes. None of the selected studies focused on the disease or area of child and adolescent mental health and mental health in general. Except for three [25, 29, 30], ten studies focused on an individual disease or area; few covered multiple diseases and the broader medical field (Table 1).

**Table 1:** Comparative analysis of extracted information from the included studies

Paper	Disease/Area	Date	Sample Size	Tool & Technology	Algorithms	Data Processing	Cohort	Utility
[18]	Prostate cancer	2010–2018	5461 patients	NLTK [33]	SVM [34], Rule-based algorithms, ConText [35], NegEx [36]	Imputation, Vectorization	Early-stage cancer patients	Clinical & pathological TNM staging
[19]	Ophthalmology	2013–2016	286 visits	R [37], Mobile devices, Numbers [38]	Bespoke Algorithms	Data drop	Ophthalmology outpatient	Clinical workflow analysis
[20]	Ophthalmology	2015–2016	8,703 visits	R 3.4.3 [37]	Linear regression [39], RF [40]	Rule And Condition	Pediatric ophthalmology outpatient	Outpatient visit length
[21]	Non-small cell lung cancer	2010–2018	794 patients	Scikit-Learn 0.24.1 [41], LightGBM 3.2.0 [42], SciPy 1.6.2 [43], BERT [44]	Logistic regression [45], RF [40], SVM [34], Deep neural network [46]	NER, Rule-Based, NLP Relation Classification, Postprocessing Modules	CT-scanned non-small cell lung cancer patients	Preoperative prediction of lymph node metastasis
[22]	Type II diabetes	-	997 patients	Python 3.6, PyTorch 1.0 [47], NVIDIA Titan X GPU, CUDA 9.0 [48], PyPhewas [49]	ADAM [50], 3D UNet [51], Fuzzy C means [52], Convolutional neural network	Segmentation & Slicing, Feature Extraction & Normalization, Annotation	CT scanned patients with and without diabetes	Early Detection of type II diabetes mellitus
[23]	Acute ischemic stroke	1992–2019	6,136, 686 patients	OHDSI tool [53], R [37], OMOP CDM [54]	Lasso logistic regression [55]	Rule-based processing	Patients aged 45 + with first ischemic stroke	Early prediction of symptomatic intracerebral hemorrhage
[24]	Nasopharyngeal cancer	2008–2018	54,703 patients	-	-	ETL, Data Structurization & Normalization	Nasopharyngeal carcinoma patient receiving treatment	Platform development for retrospective clinical studies
[25]	No specific disease	1980–2014	704,587 patients	NCBO BioPortal [56], Open Biomedical Annotator [57]	RF [40], PCA [58], GMM [59], K-Means, ICA [60], Multi-Layer Neural Network [46], LDA [61], SDA [62], NegEx [36]	Denoising, Topic Modelling, Negation	Patients with one recorded ICD code	Onset of disease based on EHRs
[26]	Cancer	1996–2012	7000 reports	Weka Software 3.6.11 [63], Perl Lingua Stem module [64], SAS 9.4 [65], MetaMap [66]	Logistic regression [45], Naive Bayes [67], K–NN [68], RF [40], J48 decision tree [69], NegEx [36]	Kullback-Leibler [70], NER, Dictionary and Non-dictionary approach, Rule-based classifier	Patients with a recorded clinical note	Detect cancer cases using plaintext medical data
[27]	Inpatient Accidental Falling	2010–2014	46,241 patients	Ubuntu 14.04 LTS [71], R 3.1.2 [37], lme4 package [72], Epi [73]	Multilevel Logistic Regression [74]	Transformation, Mapping Values	Hospitalized inpatients with recorded data	Predict fall risk to prevent injury
[28]	Pediatric Care	2008–2013	149,604 visits	Excel 2010 [75], Access 2010 [76]	-	Statistical Analysis, Correlation, Interpolation	Pediatric physician visits	Compute physician & departmental performance
[29]	No specific: Evaluated in Colorectal Cancer	-	*20346 visits	LinkEHR [77, 78], XML [79], Semantic tool [80], Saxon [81], OWL [82], NCBO BioPortal [56], Protégé [83], Hermit Reasoner [84], UMLS [85], OpenEHR [86], SNOMED CT [87], SPARQL [88]	Bespoke phenotyping algorithm, Ontology mapping, Semantic Reasoning	Semantic Representation, Standardization	Colorectal cancer patients	Identification of patient cohorts
[30]	No specific: Evaluated in HIV, hepatitis C, lab measurements	-	**Multiple	CogStack [89], Bio-YODIE [90], Elasticsearch [91], UMLS [92, 93], SPARQL [88], SNOMED CT [87]	Bidirectional recurrent neural network [94]	NER, Normalization, Semantic Indexing & Computation Negation, Indexing	Pertinent clinical notes for target use cases	Customized care, trial recruitment, and research

* = Not explicitly reported: Approximately; ** = 100 patient from MIMIC-III [95] for lab measurement, 200 and 1000 CRIS [96] patients for hepatitis C, HIV respectively

Generally, individual disease-focused studies are easier to conduct and have higher clinical utility and applicability, but they lack generalizability and have a risk of bias. Seven of the studies used data for five or more years. The longer the data collection period, the more likely it is to obtain a more accurate estimate of the incidence rate, associated factors, and prognosis; however, it increases cost and diminishes variability. Except for the four [19, 21, 22, 26], nine studies had a sample size of thousands or more (Table 1). Results also show that the relationship between data processing methods, tools, technologies, algorithms, and utility is not straightforward.

**Fig. 3:**
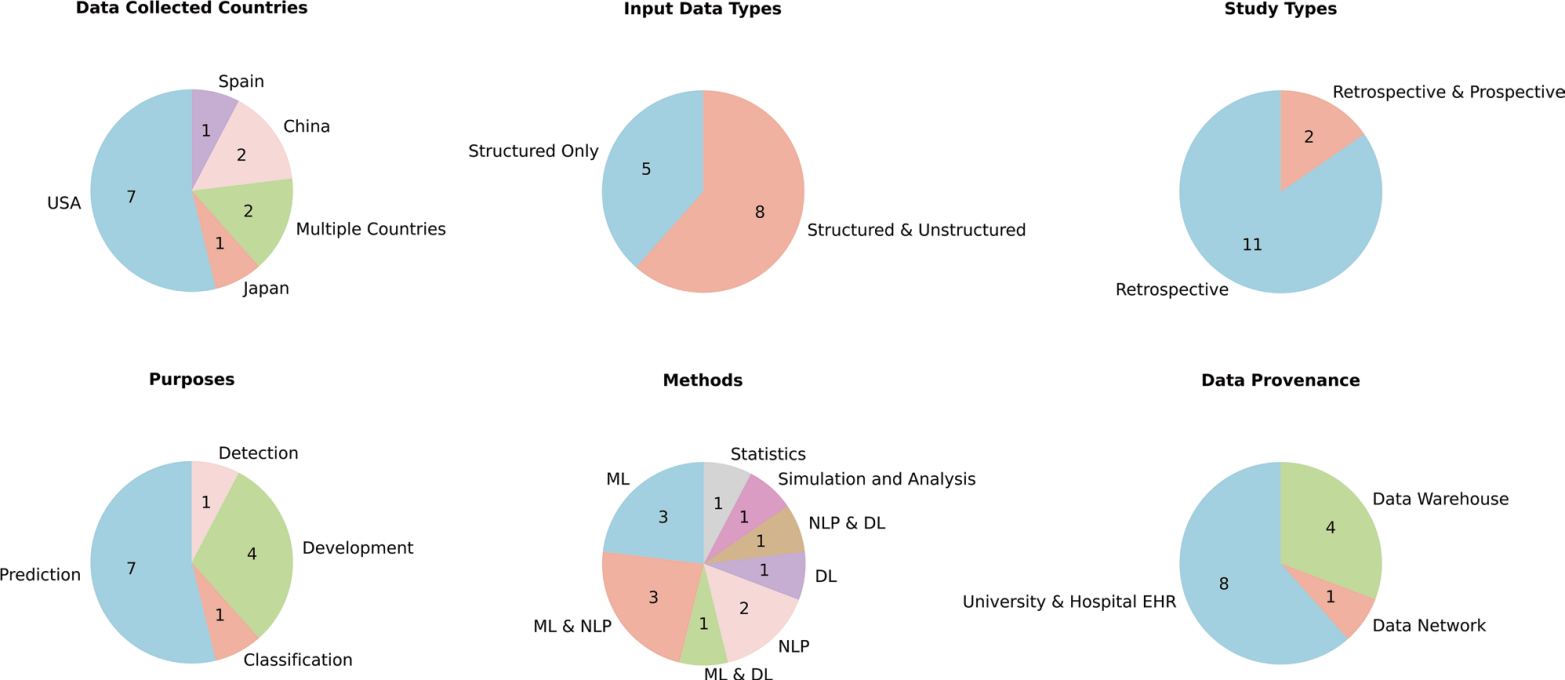
Categorization based on country of data collection, data type, study type, purpose, methods and provenance from the included studies

Figure 3 below categorizes the included studies based on country, data type, study type, purpose, methods, and provenance. It shows secondary use of EHRs was observed in developed countries: the United States of America (USA), China, Spain, Japan, Germany, and the United Kingdom (Fig. 3). The majority of secondary use studies used both structured and unstructured data. These data can be a range of forms: patient registries, demographics, text, images, voice, video, financial and insurance data, invoices, and scanned handwritten notes [97]. Most of the included studies were retrospective, with a few being a mixture of prospective and retrospective. Secondary use can be for a variety of purposes [19, 97–99] while we observed it was most commonly used for prediction [19–23, 25, 27], classification [18], detection [26], and development-related tasks and studies [24, 28–30]. For secondary uses, Machine Learning (ML) [20, 23, 27], Deep Learning (DL) [22], and Natural Language Processing (NLP) [26, 29], and their combinations [18, 21, 24, 25, 30] were observed to be applied. NLP, especially in the case of unstructured text and in structured text is the norm [26, 29]. DL was used for more extensive and multimodal data [22]. For some straightforward tasks, statistics, simulation, and statistical analysis were found to be used [19, 28]. Data provenance is where and how the data came from in the database. Hospital healthcare information systems collected most of the data [18–22, 24, 27, 30]. Others were stored and retrieved from the data warehouse of a larger healthcare information system in a larger region [25–29]. One was from the Observational Health Data Sciences and Informatics (OHDSI) data network [23].

**Fig. 4:**
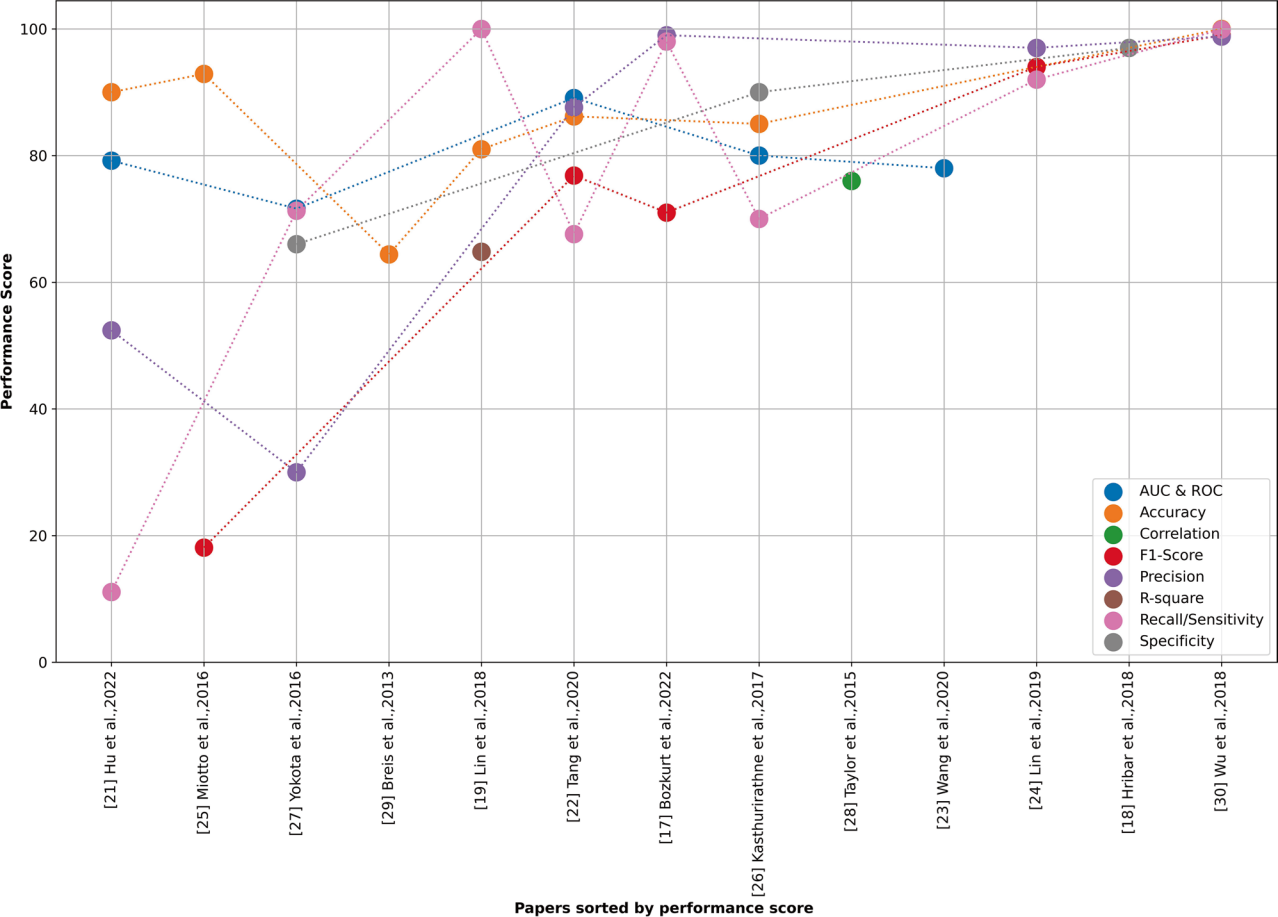
Plot of performance measures with highest values on included studies

Figure 4 below shows the performance measures in each included study. However, no conclusion can be drawn as to why some studies received higher scores than others, as there are so many factors (Fig. 4). Comparatively, it can be perceived that in studies [21, 25, 27], which were prediction-related studies using large or high sample sizes and used DL, NLP achieved lower performance than others. Moreover, lower sample size studies also obtained high performances [19, 22, 26], which is kind of mostly obvious because fewer samples lead to fewer outliers, are manageable, and easy to analyze. Figure 4 does not clearly indicate the circumstances under which performance measures such as sensitivity/recall, specificity, and correlation should be employed in comparison to other performance measures. Nevertheless, it is notable that studies employing sensitivity/recall, specificity, and correlation measures were related to statistical analysis. The standard is for simple and easy computation, correlation, specificity, and sensitivity to be used to evaluate analytical procedure performance [100].

## Discussion

The review demonstrated that secondary use has the potential to benefit a larger population in a cost effective, time and resource efficient manner. This review focused on studies directly contributing to secondary use, excluding those only mediating through statistical and secondary analysis.

The initial step was identifying appropriate topics, keywords, and inclusion and exclusion criteria, which was extremely important and challenging. The team refined the title, keywords, objective, inclusion, and exclusion criteria through three iterative meetings. The review focused on the alignment of secondary use for clinical utility. It emphasized diagnosis, detection, identification, recognition, treatment, prediction, prognosis, progress, development, onset, assessment, management, excluding secondary analysis.

It was observed that none of the studies focused on the secondary use of continuously growing datasets and systems that work well in completely unseen datasets. The EHRs used were secondary, as data provenance indicated that the collection intent was to accumulate records by the healthcare system in universities, hospitals, a region, and a research data network (Fig. 3). Information extraction is crucial in the review for comparing and understanding what and how questions. This extraction was based on topics, keywords, inclusion-exclusion criteria, and consultation with all authors involving humans and automation tools. The effectiveness of automation tools could have been improved. A substantial quantity of information was retrieved to ensure no important information was overlooked. This was further distilled, categorized, and narrowed based on comparison and relevance to the topic, as reflected in the results section above.

None of the selected studies were on mental health, but it does not affect the review as it is about the secondary use of EHRs in general, not specific to mental health. This review accessed information in higher dimensions, including information about the analysis, variables used, clinical setting, sample size, outcome measured, and others (Fig. 3, Table 1). Most studies mentioned the ethical approval from a responsible authority [18–21, 24, 27, 28], which should be and is necessary. Secondary use and ultimate utility are related, as in all included studies, the secondary use of data was utility-driven, but they did not emphasize the secondary use of continuously growing, unseen data sets for systems that can continuously learn, compute, and improve. For this, one possible technology could be a proactive training method that allows continuous training of Artificial Intelligence (AI) models using mini-batch stochastic gradient descent (SDG) and also addresses concept drift [101]. On the other hand, statistical analysis is essential to get valuable insight into the data to fully understand a problem or phenomenon. It provides insight regardless of whether the data is primary or secondary. It involves taking existing data collected for different primary purposes, performing analysis, finding new questions and insights, not necessarily focusing on direct utility, and relying mainly on statistical methods and analytical techniques. In all health informatics studies, data can be of any type, structured, unstructured, or combinations of both; it does not matter much; however, structured data are easy to use and understand. In contrast, unstructured data have a higher probability of providing more information.

From the included studies, we observed that the secondary use of EHRs for prediction, detection, and treatment in clinical decision support systems provided geographic and socioeconomic insights and demonstrated associations with utility, application, data processing, and system development. However, there are challenges related to data handling, data diversity, and ethical and regulatory considerations. Some aspects may change over time, and future developments are needed. All these issues are discussed below.

### Geographical and socio-economic insight

Technological development and adaptation have caused the explosion of healthcare data all around the world, with a projected compounded growth rate of 36% [102]. This shows that the need to collect data for research could be minimized in most cases as it is already collected in EHRs, information systems associated with a hospital, data networks, and data warehouses of regional healthcare networks. Upon analyzing the countries of the studies, it was observed that secondary use happens in developed and rich countries. However, it does not mean it is not essential and possible in developing or least developed countries. It reflects that developed countries have better healthcare infrastructure systems, leading innovation and research from the front. Other countries may increase secondary use once they are well-equipped and prepared.

### Utility and application

Included studies collected data for administrative purposes, research, and clinical observations, but they were repurposed for a different utility and application. The principal observed utilities were prediction, detection, classification, platform development for clinical decision support services, treatment recommendation, and assistance. It was observed most medical research involves secondary use of data, whereas the use of terminology secondary use or observational data is not prevalent, which is necessary and important for reliability, validity, and making review more informative. The utility and application of a given research project depend on a number of factors, including the research focus such as specific disease or multiple diseases, data collection period, sample size, identification of an appropriate cohort, data processing, tools employed, ethical and regulatory rules, and the geographical and socioeconomic conditions. The secondary use research focusing on a specific disease rather than more general or covering multiple diseases may have better performance and higher clinical utility than the opposite.

### Challenges and approaches in data handling

These data could be of sub-optimal and sometimes of poorer quality and may not be appropriate for clinical utility purposes, so it is recommended to check the completeness, breadth, density, and ability to predict [103] before proceeding further. Also, the presence of different types of data poses a significant challenge, as many of these EHRs contain potentially personally identifiable information that can be re-identified even after de-identification [97]; with today’s technology, however, these must be and can be minimized. Diverse EHRs pose a challenge, necessitating substantial effort in data processing and standardization. Despite these challenges, the varied nature of these records offers valuable opportunities for different mining and analytics applications [104]. It should be noted that none of the included studies were solely prospective, as they are not suitable for secondary uses. In contrast, retrospective and a combination of retrospective and prospective studies are used. As shown in the results above, most secondary use involved structured data, primarily ML, NLP, and DL, which may present certain challenges. Conversely, few used pure statistics, simulation, and analysis (Fig. 3).

### Ethical and regulatory considerations

Privacy and ethical concerns are the first and most important steps in using EHRs. It includes informed consent from the patient and approval from internal and external review boards. This concern has been improved through legislation, regulation, de-identification, anonymization, and pseudonymization techniques. General Data Protection Regulation (GDPR) prohibits EHRs use unless they are in the public interest, for improving health care, or for specific scientific and research purposes and fulfill their criteria [105]. Health Insurance Portability and Accountability Act (HIPAA) restricts the use and disclosure of protected health information (PHI) [106]. The recently introduced Digital Personal Data Protection Act [107] in India mentions the use of EHRs only for specific, clear, lawful purposes. Fairly and reasonably, respecting the data subject’s privacy. This is a matter of importance to all nations, and it is regulated. The secondary use necessitates addressing privacy and ethical concerns to ensure safety, quality, system development, evaluation, and maintenance.

### Data processing and system development

Data quality assessment methods, technologies, and tools are useful for data quality, completeness, comprehensiveness, and usefulness checking [108]. Data integration, data source federation, preprocessing, transformation, extraction, and mining are essential to manage healthcare data. Tools such as PowerCenter [109], Tableau [110], and Hadoop [111] in healthcare [112] are widely used in healthcare for data integration and analysis. Microsoft Excel [75] and PowerBI [113] are common for data visualization and reporting. The Common Data Model (CDM) [114] facilitates standardized data formats and interoperability. Data federation tools by Oracle [115], SAP [116], and IBM [117] support integrating data from diverse sources, ensuring that different data systems can work together seamlessly. Preprocessing and mining tools like RapidMiner Studio [118], KNIME Analytics Platform [119], and Weka [63] are used to clean, transform, and analyze data [120–122].

For the purposes of secondary use system development standards such as Informatics for Integrating Biology and the Bedside (i2b3) [123], tranSMART [124], and openEHR [125] focus on data storage and management. Standards for data transfer includes Fast Healthcare Interoperability Resources (FHIR) [126], Health Level Seven (HL7) [127], and Digital Imaging and Communications in Medicine (DICOM) [128]. Collaboration consortia patient centered outcomes research institute’s PCORnet [129] and these standards facilitate the seamless exchange of information, enabling more comprehensive data analysis and improving the utility of integrated EHRs.

Usually, secondary use system development incorporates the whole process from data quality assessment to deployment and maintenance. System integration, interoperability, ease of use, maintainability, scalability, programming languages used, and other software engineering activities are equally important. However, the main goal of secondary use is clinical utility, enhancing the effectiveness of data-driven decision-making in healthcare and improving healthcare.

### Implication and future

With time, the tools and technology will evolve. For example, AI, multimodal large language models [130], transformers [131], meta-learning, and few-shot learning [132] are just a few of the emerging technologies that will shape the future of the use of AI in CDSSs. Additionally, quantum computing, different algorithms, programming languages, and many other breakthroughs are underway. Most studies applied unsupervised and supervised techniques, reflecting AI’s effectiveness in utilizing EHRs. Self-supervised learning [133] which generates required labels without requiring human annotation and external supervision, meta-learning learning to learn ability [132] can be a game changer for representation learning [134], data augmentation in data scarcity [135], medical condition detection [136]. However, these were not clearly observed when analyzing the included studies. Consequently, the researchers and practitioners should carefully select suitable tools, technologies, data processing methods, and algorithms, ensuring they align with the final objective, context, data type, utility, and study. Also, the ethical and legal aspects, privacy, consent management, data quality assurance, transparency, and the health and life of people should always be considered. The appropriate secondary use of EHRs with proactive training has immense potential for making healthcare efficient and universal and meeting the demands of the ever-growing population, data, and healthcare challenges.

## Conclusion

Questions and problems drive the secondary use of EHRs. However, they can also be data-driven, which does not necessitate questions or hypotheses to be defined beforehand and the stockpiled data can guide what is possible. The steps for accessing and utilizing secondary use of EHRs begin with verifying that the records are sufficiently comprehensive, detailed, broad, and predictive. It is essential to perform statistical data analysis and data preprocessing as needed. Then, let the data and practicality guide the methods and techniques, and proactive training makes computation continuous.

Finally, it is crucial to assess performance across several dimensions. Secondary use can improve both somatic and mental healthcare through analysis, research, quality and safety assessment, economics, decision making, and many other uses, including commercial and non-commercial. From a clinical and patient perspective, the most important is the direct clinical benefit to patients and clinicians and the potential for clinical decision support. This includes consideration of background clinical knowledge guidelines and facts about medicine, systems, physiology, biology, nature, models, humans, and causality. Hence, moral, societal, and human dimensions are also of paramount importance.

## Electronic supplementary material

Below is the link to the electronic supplementary material.

Supplementary Material 1

## Data Availability

All data used to support the conclusions are presented and included in the article.

## List of Abbreviations

AI: Artificial Intelligence
BERT: Bidirectional Encoder Representations from Transformers
CAMH: Child and Adolescent Mental Health
CDM: Common Data Model
CDSS: Clinical Decision Support System
CRIS: Clinical Record Interactive Search
CT: Computed Tomography
DICOM: Digital Imaging and Communications in Medicine
DL: Deep Learning
ETL: Extract, Transform, and Load
FHIR: Fast Healthcare Interoperability Resources
GDPR: General Data Protection Regulation
GPU: Graphics Processing Unit
HIPAA: Health Insurance Portability and Accountability Act
HIV: Human Immunodeficiency Virus
HL7: Health Level Seven
i2b3: Integrating Biology and the Bedside
ICD: International Classification of Diseases
ICD: International Classification of Diseases
IDDEAS: Individualized Digital DEcision Assist System
ICA: Independent Component Analysis
KNN: K-Nearest Neighbors
LDA: Latent Dirichlet Allocation
ML: Machine Learning
NER: Named Entity Recognition
NLP: Natural Language Processing
NLTK: Natural Language Toolkit
OHDSI: Observational Health Data Sciences and Informatics
OMOP: Observational Medical Outcomes Partnership
OWL: Web Ontology Language
PCA: Principal Component Analysis
PHI: Protected Health Information
PRISMA: Preferred Reporting Items for Systematic Reviews and Meta-Analyses
PROSPERO: International Prospective Register of Systematic Reviews
RF: Random Forest
SDA: Stacked Denoising Autoencoders
SDG: Stochastic Gradient Descent
SNOMED CT: Systematized Nomenclature of Medicine Clinical Terms
SPARQL: SPARQL Protocol and RDF Query Language
SVM: Support Vector Machine
TNM: Tumor, Node and Metastasis
UMLS: Unified Medical Language System
WHO: World Health Organization
XML: Extensible Markup Language

## References

[1] Ehrenstein V, et al. Obtaining Data from Electronic Health Records, in Tools and Technologies for Registry Interoperability, Registries for Evaluating Patient Outcomes: a User’s Guide, 3rd Addendum 2 [Internet]. 2019. Agency for Healthcare Research and Quality (US). 31891455

[2] Tang PC, et al. Personal health records: definitions, benefits, and strategies for overcoming barriers to adoption. J Am Med Inf Assoc. 2006;13:121–26. 10.1197/jamia.M2025 PMC1447551 16357345

[3] Vest JR, Gamm LD.Health information exchange: persistent challenges and new strategies. J Am Med Inform Assoc: JAMIA. 2010;17(3):288. 20442146 10.1136/jamia.2010.003673 PMC2995716

[4] Häyrinen K, Saranto K, Nykänen P.Definition, structure, content, use and impacts of electronic health records: a review of the research literature. Int J Med Inform. 2008;77(5):291–304. 17951106 10.1016/j.ijmedinf.2007.09.001

[5] Colombo F, Oderkirk J, Slawomirski L. Health information systems, electronic medical records, and big data in global healthcare: progress and challenges in oecd countries. Handbook Glob Health. 2020;1–31.

[6] WHO. Meeting on secondary use of health data 2022 (23 July 2023); Available from: https://www.who.int/europe/news-room/events/item/2022/12/13/default-calendar/meeting-on-secondary-use-of-health-data.

[7] Shah SM, Khan RA. Secondary Use of Electronic Health Record: opportunities and Challenges. vol. 8. IEEE access; 2020. p. 136947–65.

[8] Lewkowicz D, Wohlbrandt A, Boettinger E. Economic Impact of Clinical Decision Support Interventions Based on Electronic Health Records. vol. 20. BMC Health Services Research; 2020. p. 1–12. 10.1186/s12913-020-05688-3 PMC7491136 32933513

[9] Romano MJ, Stafford RS.Electronic health records and clinical decision support systems: impact on national ambulatory care quality. Arch Intern Med. 2011;171(10):897–903. 21263077 10.1001/archinternmed.2010.527 PMC4016790

[10] Weiner SJ, et al. Effect of electronic health record clinical decision support on contextualization of care: a randomized clinical trial. JAMA Netw Open 2022;5:e2238231–e2238231. 36279133 10.1001/jamanetworkopen.2022.38231 PMC9593230

[11] Hollis C, et al. Technological innovations in mental healthcare: harnessing the digital revolution. Br J Psychiatry 2015;206:263–65. 25833865 10.1192/bjp.bp.113.142612

[12] Berner ES, La Lande TJ. Overview of clinical decision support systems. Clinical decision support systems: theory and practice. 2016:1–17.

[13] Musen MA, Middleton B, Greenes RA. Clinical Decision-support Systems, in Biomedical Informatics: computer Applications in Health Care and Biomedicine. Springer, 2021:795–840.

[14] Agency’s EM GDPR and the secondary use of health data Report from EMA workshop held with the EMA Patients’ and Consumers’ Working Party (PCWP) and Healthcare Professionals’ Working Party (HCPWP) 2020 (20 August 2023); Available from: https://www.ema.europa.eu/en/documents/report/report-workshop-application-general-data-protection-regulation-gdpr-area-health-and-secondary-use-data-medicines-and-public-health-purposes_en.pdf.

[15] Cheng HG, Phillips MR. Secondary analysis of existing data: opportunities and implementation. Shanghai archives of psychiatry. 2014;26(6):371. 10.11919/j.issn.1002-0829.214171 PMC4311114 25642115

[16] Clausen CE, et al. Testing an individualized digital decision assist system for the diagnosis and management of mental and behavior disorders in children and adolescents. BMC Med Inf Decis Making. 2020;20:1–9. 10.1186/s12911-020-01239-2 PMC7500554 32943029

[17] Page MJ, et al. The PRISMA 2020 statement: an updated guideline for reporting systematic reviews. Bmj. 2021;372. 10.1136/bmj.n71 PMC8005924 33782057

[18] Bozkurt S, et al. Expanding the secondary use of prostate cancer real world data: automated classifiers for clinical and pathological stage. Front Digit Health. 2022;4:793316. 35721793 10.3389/fdgth.2022.793316 PMC9201076

[19] Hribar MR, et al. Secondary use of electronic health record data for clinical workflow analysis. J Am Med Inf Assoc 2018;25:40–46. 10.1093/jamia/ocx098 PMC6080808 29036581

[20] Lin W-C, et al. Secondary use of electronic health record data for prediction of outpatient visit length in ophthalmology clinics. in AMIA Annual Symposium Proceedings. 2018. American Medical Informatics Association. PMC6371379 30815183

[21] Hu D, et al. Using natural language processing and machine learning to preoperatively predict lymph node metastasis for non–small cell lung cancer with electronic medical records: development and validation study. JMIR Med Inform 2022;10:e35475. 35468085 10.2196/35475 PMC9086872

[22] Tang Y, et al. Prediction of type II diabetes onset with computed tomography and electronic medical records. In Multimodal Learning for Clinical Decision Support and Clinical Image-Based Procedures: 10th International Workshop, ML-CDS 2020, and 9th International Workshop, CLIP 2020, Held in Conjunction with MICCAI 2020, Lima, Peru, October 4–8, 2020, Proceedings 9. 2020. Springer. 10.1007/978-3-030-60946-7_2 PMC8188902 34113927

[23] Wang Q, et al. Development and validation of a prognostic model predicting symptomatic hemorrhagic transformation in acute ischemic stroke at scale in the OHDSI network. PLoS One 2020;15:e0226718. 31910437 10.1371/journal.pone.0226718 PMC6946584

[24] Lin L, et al. Development and implementation of a dynamically updated big data intelligence platform from electronic health records for nasopharyngeal carcinoma research. Br J Radiol 2019;92:20190255. 31430186 10.1259/bjr.20190255 PMC6774598

[25] Miotto R, et al. Deep patient: an unsupervised representation to predict the future of patients from the electronic health records. Sci Rep 2016;6:1–10. 27185194 10.1038/srep26094 PMC4869115

[26] Kasthurirathne SN, et al. Toward better public health reporting using existing off the shelf approaches: the value of medical dictionaries in automated cancer detection using plaintext medical data. J Biomed Informat. 2017;69:160–76. 10.1016/j.jbi.2017.04.008 28410983

[27] Yokota S, Ohe K.Construction and evaluation of FiND, a fall risk prediction model of inpatients from nursing data. Japan J Nurs Sci. 2016;13(2):247–55. 27040735 10.1111/jjns.12103

[28] Taylor B, MacPhee S. Physician and Departmental Performance Metrics in Pediatric Emergency Care: secondary Use of Patient Visit Data. vol. 63. Procedia Computer Science; 2015. p. 190–97.

[29] Fernández-Breis JT, et al. Leveraging electronic healthcare record standards and semantic web technologies for the identification of patient cohorts. J Am Med Inf Assoc 2013;20:e288–e296. 10.1136/amiajnl-2013-001923 PMC3861938 23934950

[30] Wu H, et al. SemEHR: a general-purpose semantic search system to surface semantic data from clinical notes for tailored care, trial recruitment, and clinical research. J Am Med Inf Assoc 2018;25:530–37. 10.1093/jamia/ocx160 PMC6019046 29361077

[31] Ouzzani M, et al. Rayyan—a web and mobile app for systematic reviews. Systematic reviews. 2016;5:1–10. 10.1186/s13643-016-0384-4 PMC5139140 27919275

[32] Ought. Elicit: The AI Research Assistant. 2023 (22 February 2023); Available from: https://elicit.org.

[33] Bird S, Klein E, Loper E. Natural Language Processing with Python: analyzing Text with the Natural Language Toolkit. “O’Reilly Media, Inc.”; 2009.

[34] Cortes C, Vapnik V. Support-vector networks. Machine Learning. 1995;20:273–97.

[35] Harkema H, et al. ConText: an algorithm for determining negation, experiencer, and temporal status from clinical reports. J Biomed Informat 2009;42:839–51. 10.1016/j.jbi.2009.05.002 PMC2757457 19435614

[36] Chapman WW, et al. A simple algorithm for identifying negated findings and diseases in discharge summaries. J Biomed Informat 2001;34:301–10. 10.1006/jbin.2001.1029 12123149

[37] Team RC, R: a language and environment for statistical computing.

[38] Inc., A.. Apple. Numbers. 2021.

[39] James G, et al. An Introduction to Statistical Learning, vol. 112. Springer. 2013.

[40] Breiman L. Random forests. Machine Learning. 2001;45:5–32.

[41] Pedregosa F, et al. Scikit-learn: machine learning in Python. J Mach Learn Res. 2011;12:2825–30.

[42] Ke G, et al. Lightgbm: a highly efficient gradient boosting decision tree. Adv Neural Inf Process Syst. 2017;30.

[43] Virtanen P, et al. SciPy 1.0: fundamental algorithms for scientific computing in Python. Nat Methods 2020;17:261–72. 32015543 10.1038/s41592-019-0686-2 PMC7056644

[44] Devlin J, et al., Bert: pre-training of deep bidirectional transformers for language understanding. arXiv preprint arXiv:1810.04805, 2018.

[45] Hosmer Jr DW, Lemeshow S, Sturdivant RX. Applied Logistic Regression. John Wiley & Sons; 2013.

[46] LeCun Y, Bengio Y, Hinton G. Deep learning. nature. 2015;521(7553):436–44. 10.1038/nature14539 26017442

[47] Paszke A, et al. Pytorch: an imperative style, high-performance deep learning library. Adv Neural Inf Process Syst. 2019;32.

[48] Nvidia, CUDA. 2006.

[49] Chaganti S, et al. Contextual deep regression network for volume estimation in orbital CT. In Medical Image Computing and Computer Assisted Intervention–MICCAI 2019: 22nd International Conference, Shenzhen, China, October 13–17, 2019, Proceedings, Part VI 22. 2019. Springer. 10.1007/978-3-030-32226-7_12 PMC8796819 35098262

[50] Kingma DP, Ba J, Adam: a method for stochastic optimization. arXiv preprint arXiv:1412.6980, 2014.

[51] Çiçek Ö, et al. 3D U-Net: learning dense volumetric segmentation from sparse annotation. In Medical Image Computing and Computer-Assisted Intervention–MICCAI 2016: 19th International Conference, Athens, Greece, October 17-21, 2016, Proceedings, Part II 19. 2016. Springer.

[52] Bezdek JC, Ehrlich R, Full W. FCM: the fuzzy c-means clustering algorithm. Computers & geosciences. 1984;10(2-3):191–203.

[53] Hripcsak G, et al. Observational Health Data Sciences and Informatics (OHDSI): opportunities for observational researchers. Stud Health Technol Inform. 2015;216:574. 26262116 PMC4815923

[54] OHDSI. Observational health data sciences and informatics. OMOP Common Data Model. 2014.

[55] Tibshirani R.Regression shrinkage and selection via the lasso. J R Stat Soc Ser B Stat Method. 1996;58(1):267–88.

[56] Musen MA, et al. The national center for biomedical ontology. J Am Med Inf Assoc 2012;19:190–95. 10.1136/amiajnl-2011-000523 PMC3277625 22081220

[57] Jonquet C, Shah NH, Musen MA. The open biomedical annotator. Summit on Trans Bioinform. 2009;2009:56. PMC3041576 21347171

[58] Gewers FL, et al. Principal component analysis: a natural approach to data exploration. ACM Computing Surveys (CSUR) 2021;54:1–34.

[59] Reynolds DA. Gaussian mixture models. Enc Biometrics. 2009;741:659–63.

[60] Hyvärinen A, Oja E.Independent component analysis: algorithms and applications. Neural Netw. 2000;13(4–5):411–30. 10946390 10.1016/s0893-6080(00)00026-5

[61] Blei DM, Ng AY, Jordan MI.Latent dirichlet allocation. J Mach Learn Res. 2003;3(Jan):993–1022.

[62] Vincent P, et al. Stacked denoising autoencoders: learning useful representations in a deep network with a local denoising criterion. J Mach Learn Res. 2010;11(12).

[63] Hall M, et al. The WEKA data mining software: an update. ACM SIGKDD Explorations Newsletter. 2009;11(1):10–18.

[64] Estudillo-Valderrama MA, et al. A distributed approach to alarm management in chronic kidney disease. IEEE J Biomed Health Inform 2014;18:1796–803. 25014977 10.1109/JBHI.2014.2333880

[65] Institute S. SAS/STAT® online documentation, Version 9.4.

[66] Aronson AR, Lang F-M.An overview of MetaMap: historical perspective and recent advances. J Am Med Inf Assoc. 2010;17(3):229–36. 10.1136/jamia.2009.002733 PMC2995713 20442139

[67] McCallum A, Nigam K. A comparison of event models for naive bayes text classification. In AAAI-98 workshop on learning for text categorization. 1998. Madison, WI.

[68] Cover T, Hart P.Nearest neighbor pattern classification. IEEE Trans Inf Theory. 1967;13(1):21–27.

[69] Quinlan JR. C4. 5: programs for Machine Learning. Elsevier; 2014.

[70] Polani D, et al Kullback-Leibler Divergence. In Dubitzky W. editor Encyclopedia of Systems Biology. New York: Springer New York. 2013: 1087–88.

[71] Ltd C. Ubuntu 14.04.6 LTS (Trusty Tahr). 2014.

[72] Bates D, et al., lme4: linear mixed-effects models using Eigen and S4. R package version 1.1-7. 2014.

[73] Bendix Carstensen MP, Laara E, Hills M, Epi: a package for statistical analysis in epidemiology.

[74] Gelman A, Hill J. Data Analysis Using Regression and Multilevel/hierarchical Models. Cambridge university press; 2006.

[75] Microsoft. Microsoft Excel.

[76] Microsoft. Microsoft Access

[77] Maldonado JA, et al. Using the ResearchEHR platform to facilitate the practical application of the EHR standards. J Biomed Informat. 2012;45:746–62. 10.1016/j.jbi.2011.11.004 22142945

[78] Maldonado JA, et al. LinkEHR-Ed: a multi-reference model archetype editor based on formal semantics. Int J Med Inform. 2009;78:559–70. 19386540 10.1016/j.ijmedinf.2009.03.006

[79] W3C. XQuery 1.0: An XML Query Language 2010 (cited 2023 20 August); Available from: https://www.w3.org/TR/xquery.

[80] sele.inf.um.es. Semantic Web Integration Tool (SWIT) Available from: http://sele.inf.um.es/swit/.

[81] Saxonica. Saxon XSLT and XQuery processor. Available from: https://www.saxonica.com/welcome/welcome.xml.

[82] Group, W.C.O.W. OWL 2 web ontology language document overview. Available from: https://www.w3.org/TR/owl2-overview/.

[83] Musen MA.The protégé project: a look back and a look forward. AI Matters. 2015;1(4):4–12. 27239556 10.1145/2757001.2757003 PMC4883684

[84] Shearer RD, Motik B, Horrocks I. Hermit: a highly-efficient OWL reasoner. Owled. 2008.

[85] Medicine NLO UMLS Terminology Services [cited 2023 27 August]; Available from. https://uts.nlm.nih.gov/uts/.

[86] Foundation, o. openEHR Clinical Knowledge Manager [cited 2023 27 August]; Available from: https://ckm.openehr.org/ckm/.

[87] International S. SNOMED Software and Tools. [cited 2023 27 August]; Available from: https://www.snomed.org/software-tools.

[88] International S. SPARQL Query Language for RDF. [cited 2023 27 August]; Available from: https://www.w3.org/TR/rdf-sparql-query/.

[89] Jackson R, et al. CogStack-experiences of deploying integrated information retrieval and extraction services in a large National Health Service Foundation Trust hospital. BMC Med Inf Decis Making. 2018;18:1–13. 10.1186/s12911-018-0623-9 PMC6020175 29941004

[90] Gorrell G, Song X, Roberts A, Bio-yodie: a named entity linking system for biomedical text. arXiv preprint arXiv:1811.04860, 2018.

[91] Company ETSA Elasticsearch: the official distributed search & analytics engine. [cited 2023 27 August].

[92] Bodenreider O. The unified medical language system (UMLS): integrating biomedical terminology. Nucleic Acids Research. 2004;32(suppl_1):D267–D270. 10.1093/nar/gkh061 PMC308795 14681409

[93] Lindberg DA, Humphreys BL. The Unified Medical Language System (UMLS) and computer-based patient records. In: Aspects of the Computer-based Patient Record, Springer, 1992:165–75.

[94] Schuster M, Paliwal KK.Bidirectional recurrent neural networks. IEEE Trans Signal Process. 1997;45(11):2673–81.

[95] Johnson AE, et al. MIMIC-III, a freely accessible critical care database. Sci Data 2016;3:1–9. 10.1038/sdata.2016.35 PMC4878278 27219127

[96] Stewart R, et al. The South London and Maudsley NHS foundation trust biomedical research centre (SLAM BRC) case register: development and descriptive data. BMC Psychiatry. 2009;9:1–12. 19674459 10.1186/1471-244X-9-51 PMC2736946

[97] Safran C, et al. Toward a national framework for the secondary use of health data: an American Medical Informatics Association White Paper. J Am Med Inf Assoc 2007;14:1–9. 10.1197/jamia.M2273 PMC2329823 17077452

[98] Kosseim P, Brady M. Policy by procrastination: secondary use of electronic health records for health research purposes. McGill JL & Health. 2008;2:5.

[99] Tu K, et al. Are family physicians comprehensively using electronic medical records such that the data can be used for secondary purposes? A Canadian perspective. BMC Medical Informatics and Decision Making. 2015;15:1–12. 10.1186/s12911-015-0195-x PMC4535372 26268511

[100] Thabane L, et al. A tutorial on sensitivity analyses in clinical trials: the what, why, when and how. BMC Med Res Method. 2013;13:1–12. 10.1186/1471-2288-13-92 PMC3720188 23855337

[101] Prapas I, et al. Continuous training and deployment of deep learning models. Datenbank-Spektrum 2021;21:203–12.

[102] Coughlin S, et al. Looking to tomorrow’s healthcare today: a participatory health perspective. Internal Med J 2018;48:92–96. 29314515 10.1111/imj.13661

[103] Weiskopf NG, et al. Defining and measuring completeness of electronic health records for secondary use. J Biomed Informat 2013;46:830–36. 10.1016/j.jbi.2013.06.010 PMC3810243 23820016

[104] Sarwar T, et al. The secondary use of electronic health records for data mining: data characteristics and challenges. ACM Computing Surveys (CSUR) 2022;55:1–40.

[105] Starkbaum J, Felt U.Negotiating the reuse of health-data: research, big data, and the European general data protection regulation. Big Data Soc. 2019;6(2):2053951719862594.

[106] Cohen IG, Mello MM.HIPAA and protecting health information in the 21st century. Jama. 2018;320(3):231–32. 29800120 10.1001/jama.2018.5630

[107] Ministry of Electronics and Information Technology, G.o.I. The digital personal data protection act, 2023. 2023.

[108] Lewis AE, et al. Electronic health record data quality assessment and tools: a systematic review. J Am Med Inf Assoc 2023;30:1730–40. 10.1093/jamia/ocad120 PMC10531113 37390812

[109] Deutschland I, PowerCenter: enterprise Data Integration Platform.

[110] Tableau. Tableau: business Intelligence and Analytics Software. 2023.

[111] Foundation AS. Apache Hadoop. 2023.

[112] Nazir S, et al. Healthcare Big Data Management and Analytics in Scientific Programming. vol. 2021, Scientific Programming; 2021. 1–2.

[113] Becker LT, Gould EM.Microsoft power BI: extending excel to manipulate, analyze, and visualize diverse data. Ser Rev. 2019;45(3):184–88.

[114] Hripcsak G, et al. Characterizing treatment pathways at scale using the OHDSI network. Proc Natl Acad Sci 2016;113:7329–36. 27274072 10.1073/pnas.1510502113 PMC4941483

[115] Corporation O. Oracle| integrated Cloud 0Applications and Platform Services. 2023.

[116] SE S. SAP software solutions| business applications and technology. 2023.

[117] Corporation I. (cited 2023 27 August). Available from: https://www.ibm.com/us-en.

[118] Engineering A. RapidMiner-Best Data Science Platform for Your Enterprise (cited 2023 27 August); Available from: https://rapidminer.com/platform/.

[119] KNIME. KNIME Analytics Platform (cited 2023 27 August); Available from: https://www.knime.com/knime-analytics-platform.

[120] Tougui I, Jilbab A, El Mhamdi J.Heart disease classification using data mining tools and machine learning techniques. Health Technol. 2020;10(5):1137–44.

[121] Poucke SV, et al. Scalable predictive analysis in critically ill patients using a visual open data analysis platform. PloS One 2016;11:e0145791. 26731286 10.1371/journal.pone.0145791 PMC4701479

[122] Santos-Pereira J, Gruenwald L, Bernardino J.Top data mining tools for the healthcare industry. J King Saud Univ, Comput Inf Sci. 2022;34(8):4968–82.

[123] Murphy SN, et al. Serving the enterprise and beyond with informatics for integrating biology and the bedside (i2b2). J Am Med Inf Assoc 2010;17:124–30. 10.1136/jamia.2009.000893 PMC3000779 20190053

[124] Athey BD, et al., TranSMART: an open source and community-driven informatics and data sharing platform for clinical and translational research. AMIA Summits on Translational Science Proceedings 2013. 2013: 6. PMC3814495 24303286

[125] Beale T, Heard S.An ontology-based model of clinical information. Stud Health Technol Inform. 2007;129(1):760. 17911819

[126] build.fhir.org. FHIR v6.0.0-cibuild. (cited 2023 27 August); Available from: https://build.fhir.org.

[127] International H. Health Level Seven International - HL7. (cited 2023 27 August); Available from: https://www.hl7.org/.

[128] dicomstandard.org. DICOM Standard 2019 (cited 2023 27 August); Available from: https://www.dicomstandard.org/.

[129] The National Patient-Centered Clinical Research Network (cited 2023 27 August); Available from: https://pcornet.org/.

[130] OpenAI. GPT-4 Technical Report 2023 (cited 2023 13 September); Available from: https://cdn.openai.com/papers/gpt-4.pdf.

[131] Vaswani A, et al. Attention is all you need. Adv Neural Inf Process Syst. 2017;30.

[132] Li Z, et al., Meta-sgd: learning to learn quickly for few-shot learning. arXiv preprint arXiv:1707.09835, 2017.

[133] Balestriero R, et al., A cookbook of self-supervised learning. arXiv preprint arXiv:2304.12210, 2023.

[134] Shurrab S, Duwairi R. Self-supervised learning methods and applications in medical imaging analysis: a survey. PeerJ Comput Sci. 2022;8:e1045. 36091989 10.7717/peerj-cs.1045 PMC9455147

[135] Lemmon J, et al. Self-supervised machine learning using adult inpatient data produces effective models for pediatric clinical prediction tasks. J Am Med Inf Assoc 2023;30:2004–11. 10.1093/jamia/ocad175 PMC10654865 37639620

[136] Anton J, et al. How well do self-supervised models transfer to medical imaging? J Imaging 2022;8:320. 36547485 10.3390/jimaging8120320 PMC9782186

