# Supplementary material for: Secondary use of health records for prediction, detection, and treatment planning in the clinical decision support system: a systematic review
Source: BMC Med Inform Decis Mak. 2025 May 16;25:190. doi: 10.1186/s12911-025-03021-8 (PMC12083156; doi:10.1186/s12911-025-03021-8)
Supplement: Supplementary file 1 — Supplementary Material 1 [file 12911_2025_3021_MOESM1_ESM.docx]

# Supplementary material for "Secondary use of health records for prediction, detection, and treatment planning in the clinical decision support system: a systematic review"

Authors:

Dipendra Pant^a,d,*^, Øystein Nytrø^a,d,f^, Bennett L. Leventhal^b^, Carolyn Clausen^c^, Kaban Koochakpour^a^, Line Stien^c^, Odd Sverre Westbye^c,d^, Roman Koposov^e^, Thomas Brox Røst^h^, Thomas Frodl^g^, Norbert Skokauskas^c^

Affiliations:

^a^Department of Computer Science, Norwegian University of Science and Technology, Trondheim, Norway

^b^The University of Chicago, Chicago, Illinois, USA 
^c^Regional Centre for Child and Youth Mental Health and Child Welfare (RKBU Central Norway), Department of Mental Health, Faculty of Medicine and Health Sciences, Norwegian University of Science and Technology, Trondheim, Norway 
^d^Department of Child and Adolescent Psychiatry, Clinic of Mental Health Care, St. Olav University Hospital, Trondheim, Norway

^e^Regional Centre for Child and Youth Mental Health and Child Welfare (RKBU North), UiT The Arctic University of Norway, Tromsø, Norway 
^f^Department of Computer Science, UiT The Arctic University of Norway, Tromsø, Norway 
^g^RWTH University of Aachen, Aachen, Germany

^h^Vivit AS, Trondheim, Norway

*Corresponding Author: Dipendra Pant

1. **Supplementary Information A: Search Strategies for Each Database Based on Defined Search Terms**

| Database | Database Type | Search Terms | Search Query |
| --- | --- | --- | --- |
| PubMed | CL | AF | *((("health data" OR "health record" OR "Electronic health record" OR "Electronic medical record" OR "Observational data" OR "EHR" OR "EMR" OR "medical record") AND ("secondary use*" OR "secondary application*" OR "secondary analy*" OR "health reuse" OR "clinical reuse" OR "secondary usage")) AND ("diagnosis" OR "detect" OR "identify" OR "recognize" OR "treatment" OR "predict" OR "prognosis" OR "progress" OR "develop" OR "onset" OR "assessment"OR "management")) AND ("decision making" OR "decision-support" OR "decision support" OR "decision system" OR "computer* decision" OR "computer-aided decision" OR "computer aided decision" OR "DSS" OR "computer assisted decision making"OR "computer-assisted decision making" OR "clinical decision support*" OR "clinical-decision support*" OR CDS)* |
| Scopus | CL | TAK | *( TITLE-ABS-KEY ( "health data"  OR  "health record"  OR  "Electronic health record"  OR  "Electronic medical record"  OR  "Observational data"  OR  "EHR"  OR  "EMR"  OR  "medical record" )  AND  TITLE-ABS-KEY ( "secondary use*"  OR  "secondary application*"  OR  "secondary analy*"  OR  "health* reuse"  OR  "clinical reuse" OR “secondary usage”)  AND  TITLE-ABS-KEY ( "diagnosis"  OR  "detect"  OR  "identify"  OR  "recognize"  OR  "treatment"  OR  "predict"  OR  "prognosis"  OR  "progress"  OR  "develop"  OR  "onset"  OR  "assessment"  OR  "management" )  AND  TITLE-ABS-KEY ( "decision making"  OR  "decision-support"  OR  "decision support"  OR  "decision system"  OR  "computer* decision"  OR  "computer-aided decision"  OR  "computer aided decision"  OR  "DSS"  OR  "computer assisted decision making"  OR  "computer-assisted decision making"  OR  "clinical decision support*"  OR  "clinical-decision support*"  OR  cds ) )* |
| IEEE | CS | TAK | *("All Metadata":health data OR "All Metadata":health record OR "All Metadata":Electronic health record OR "All Metadata":Electronic medical record OR "All Metadata":Observational data OR "All Metadata":EHR OR "All Metadata":EMR OR "All Metadata":medical record) AND ("All Metadata":"secondary use*" OR "All Metadata":"secondary analy*" OR "All Metadata":"secondary application*" OR "All Metadata":"health* reuse" OR "All Metadata":clinical reuse OR "All Metadata":secondary usage) AND ("All Metadata":diagnosis OR "All Metadata":detect OR "All Metadata":identify OR "All Metadata":recognize OR "All Metadata":treatment OR "All Metadata":predict OR "All Metadata":prognosis OR "All Metadata":progress OR "All Metadata":develop OR "All Metadata":onset OR "All Metadata":assessment OR "All Metadata":management) AND ("All Metadata":decision making OR "All Metadata":decision-support OR "All Metadata":"decision support*" OR "All Metadata":"decision system*" OR "All Metadata":"computer* decision" OR "All Metadata":"computer-aided decision" OR "All Metadata":"computer aided decision" OR "All Metadata":DSS OR "All Metadata":computer assisted decision making OR "All Metadata":computer-assisted decision making OR "All Metadata":“clinical decision support*” OR "All Metadata":“clinical-decision support*” OR "All Metadata":cds)* |
| ACM DL | CS | AF | *[[All: "health data"] OR [All: "health record"] OR [All: "electronic health record"] OR [All: "electronic medical record"] OR [All: "observational data"] OR [All: "ehr"] OR [All: "emr"] OR [All: "medical record"]] AND [[All: "secondary use*"] OR [All: "secondary application*"] OR [All: "secondary analy*"] OR [All: "health reuse"] OR [All: "clinical reuse"] OR [All: " secondary usage"]] AND [[All: "diagnosis"] OR [All: "detect"] OR [All: "identify"] OR [All: "recognize"] OR [All: "treatment"] OR [All: "predict"] OR [All: "prognosis"] OR [All: "progress"] OR [All: "develop"] OR [All: "onset"] OR [All: "assessment"] OR [All: "management"]] AND [[All: "decision making"] OR [All: "decision-support"] OR [All: "decision support"] OR [All: "decision system"] OR [All: "computer* decision"] OR [All: "computer-aided decision"] OR [All: "computer aided decision"] OR [All: "dss"] OR [All: "computer assisted decision making"] OR [All: "computer-assisted decision making"] OR [All: "clinical decision support*"] OR [All: "clinical-decision support*"] OR [All: cds]]* |
| DBLP | CS | AF | Two separate searches:   1. *secondary use health data* 2. *data\|record diagnosis\|detect\|identify\|recognize\|treatment\|predict\|prognosis cdss\|clinical-decision-support* |

Database types:

- CS = Computer science
- CL = Clinical

Search terms usage in:

- Defined Search Term: 1. secondary use health data 2. data|record diagnosis|detect|identify|recognize|treatment|predict|prognosis cdss|clinical-decision-support
- TAK: Title OR Abstract OR Keywords
- AF = All fields

1. **Supplementary Information B: Search Database and Corresponding Search Strings with Results**

C.1. Scopus:

Bolk 1: "health data" OR "health record" OR "Electronic health record" OR “Electronic medical record” OR “Observational data” OR "EHR" OR "EMR" OR "medical record"

Bolk 2: "secondary use*" OR "secondary application*" OR "secondary analy*" OR "health* reuse" OR "clinical reuse" OR "secondary usage"

Bolk 3: "diagnosis"  OR "detect" OR "identify" OR "recognize" OR "treatment" OR "predict" OR "prognosis" OR "progress" OR "develop" OR "onset" OR "assessment" OR "management"

Bolk 4: "decision making" OR "decision-support" OR "decision support" OR "decision system" OR "computer* decision" OR "computer-aided decision" OR "computer aided decision" OR “DSS” OR “computer assisted decision making” OR “computer-assisted decision making” OR “clinical decision support*” OR “clinical-decision support*” OR cds

**Obtained:** 172

**Search Date:** 27 April, 2023

**Search Includes:** Title, Abstract, Keywords

C.2. IEEE Xplore

health data OR health record OR Electronic health record OR Electronic medical record OR Observational data OR EHR OR EMR OR medical record

 AND

"secondary use*" OR "secondary analy*" OR "secondary application*" OR "health* reuse" OR clinical reuse OR secondary usage

AND

diagnosis OR detect OR identify OR recognize OR treatment OR predict OR prognosis OR progress OR develop OR onset OR assessment OR management

AND

decision making OR decision-support OR "decision support*" OR "decision system*" OR "computer* decision" OR "computer-aided decision" OR "computer aided decision" OR DSS OR computer assisted decision making OR computer-assisted decision making OR “clinical decision support*” OR “clinical-decision support*” OR cds

**Obtained:** 29

**Search Date:** 27 April, 2023

**Search Includes:** *Title, Abstract, Keywords*

C.3. ACM Library:

"health data" OR "health record" OR "Electronic health record" OR “Electronic medical record” OR “Observational data” OR "EHR" OR "EMR" OR "medical record"

AND

"secondary use*" OR "secondary application*" OR "secondary analy*" OR "health* reuse" OR "clinical reuse" OR "secondary usage"

AND

"diagnosis"  OR "detect" OR "identify" OR "recognize" OR "treatment" OR "predict" OR "prognosis" OR "progress" OR "develop" OR "onset" OR "assessment" OR "management"

AND

"decision making" OR "decision-support" OR "decision support" OR "decision system" OR "computer* decision" OR "computer-aided decision" OR "computer aided decision" OR “DSS” OR “computer assisted decision making” OR “computer-assisted decision making” OR “clinical decision support*” OR “clinical-decision support*” OR cds

**Obtained:** 63

**Search Date:** 27 April, 2023

**Search Includes:** All Fields *(Title, Abstract, Keywords,Full text)*

C.4. PubMed:

"health data" OR "health record" OR "Electronic health record" OR “Electronic medical record” OR “Observational data” OR "EHR" OR "EMR" OR "medical record"

AND

"secondary use*" OR "secondary application*" OR "secondary analy*" OR "health* reuse" OR "clinical reuse"

AND

"diagnosis"  OR "detect" OR "identify" OR "recognize" OR "treatment" OR "predict" OR "prognosis" OR "progress" OR "develop" OR "onset" OR "assessment" OR "management"

AND

"decision making" OR "decision-support" OR "decision support" OR "decision system" OR "computer* decision" OR "computer-aided decision" OR "computer aided decision" OR “DSS” OR “computer assisted decision making” OR “computer-assisted decision making” OR “clinical decision support*” OR “clinical-decision support*” OR cds

**Obtained:** 83

**Search Date:** 27 April, 2023

**Search Includes:** All Fields *(Title, Abstract, Keywords,Full text)*

C.5. DBLP:

First Search: secondary use health data *(No record found using secondary usage hence removed)*

**Obtained:** 29

**Search Date:** 27 April, 2023 **Time:** 18:45

**Search Includes:** All Fields *(Title, Abstract, Keywords, Fulltext)*

Second Search: data|record diagnosis|detect|identify|recognize|treatment|predict|prognosis cdss|clinical-decision-support

**Obtained:** 15

**Search Date:** 27 April, 2023

**Search Includes:** *Title, Abstract, Keywords*

Total Obtained: 29+15 = 44
